# Algorithm for detection and screening of familial hypercholesterolemia in Lithuanian population

**DOI:** 10.1186/s12944-024-02124-x

**Published:** 2024-05-07

**Authors:** Urte Aliosaitiene, Zaneta Petrulioniene, Egidija Rinkuniene, Antanas Mainelis, Egle Brazdziuniene, Urte Smailyte, Vaida Sileikiene, Aleksandras Laucevicius

**Affiliations:** 1 Faculty of Medicine, Vilnius University, Vilnius, Lithuania; 2 Clinic of Cardiac and Vascular Diseases, Vilnius University Hospital Santaros Klinikos, Vilnius, Lithuania; 3 Faculty of Mathematics and Informatics, Vilnius University, Vilnius, Lithuania

**Keywords:** Familial hypercholesterolemia, Cascade screening, Dyslipidemia, Coronary artery disease, Genetic testing, FH-related mutations

## Abstract

**Background:**

Familial hypercholesterolemia (FH) is one of the most common autosomal dominant diseases. FH causes a lifelong increase in low-density lipoprotein cholesterol (LDL-C) levels, which in turn leads to atherosclerotic cardiovascular disease. The incidence of FH is widely underestimated and undertreated, despite the availability and effectiveness of lipid-lowering therapy. Patients with FH have an increased cardiovascular risk; therefore, early diagnosis and treatment are vital. To address the burden of FH, several countries have implemented national FH screening programmes. The currently used method for FH detection in Lithuania is mainly based on opportunistic testing with subsequent cascade screening of index cases’ first-degree relatives.

**Methods:**

A total of 428 patients were included in this study. Patients with suspected FH are referred to a lipidology center for thorough evaluation. Patients who met the criteria for probable or definite FH according to the Dutch Lipid Clinic Network (DLCN) scoring system and/or had LDL-C > = 6.5 mmol/l were subjected to genetic testing. Laboratory and instrumental tests, vascular marker data of early atherosclerosis, and consultations by other specialists, such as radiologists and ophthalmologists, were also recorded.

**Results:**

A total of 127/428 (30%) patients were genetically tested. FH-related mutations were found in 38.6% (*n* = 49/127) of the patients. Coronary artery disease (CAD) was diagnosed in 13% (*n* = 57/428) of the included patients, whereas premature CAD was found in 47/428 (11%) patients. CAD was diagnosed in 19% (*n* = 9/49) of patients with FH-related mutations, and this diagnosis was premature for all of them.

**Conclusions:**

Most patients in this study were classified as probable or possible FH without difference of age and sex. The median age of FH diagnosis was 47 years with significantly older females than males, which refers to the strong interface of this study with the LitHir programme. CAD and premature CAD were more common among patients with probable and definite FH, as well as those with an FH-causing mutation. The algorithm described in this study is the first attempt in Lithuania to implement a specific tool which allows to maximise FH detection rates, establish an accurate diagnosis of FH, excluding secondary causes of dyslipidaemia, and to select patients for cascade screening initiation more precisely.

## Introduction

Familial hypercholesterolemia (FH) is a common genetic-metabolic autosomal dominant disorder characterized by impaired metabolism of low-density lipoprotein cholesterol (LDL-C). The most common mutations that cause FH are found in the LDL receptor gene (LDLR), apolipoprotein B (APOB) and pro-protein convertase subtilisin/kexin 9 (PCSK9) genes [1, 2]. Due to the lifelong increase in blood lipoproteins, patients with FH are at increased risk of cardiovascular events, which, if left untreated, may manifest as atherosclerotic cardiovascular disease (ASCVD) [3, 4]. According to the latest data, the prevalence of FH in the general population is estimated to be approximately 1:300 and may be even greater in certain populations [5]. Furthermore, in their meta-analysis, *Behasti* et al. reported that the incidence of FH is 10-fold greater among patients with ischemic heart disease (IHD) than among the general population and is even 20-fold greater among patients with premature IHD [6]. However, studies on the prevalence of FH are lacking in most countries, including Lithuania [6]. *Petrulioniene* et al., in their previous study, estimated that there could be as many as 14 240 patients with FH in Lithuania [7]; however, the exact prevalence of the disease neither in the general population nor in the IHD population is known.

There are two ways to diagnose FH: clinical and molecular. Clinical diagnosis relies on patient phenotypic data and is usually made using standardized criteria (e.g., the Simon Broome criteria and Dutch Lipid Clinic Network criteria), which often have high specificity and low sensitivity [8, 9]. The DLCN score has been proven to be effective at detecting and evaluating FH [8]. However, evidence that clinical criteria may be outdated and less applicable than before is increasing [10]. For example, due to rapid advancements in the diagnosis and treatment of many cardiovascular diseases, some of the information may be harder to collect. Furthermore, patients may not always be tested for tendon xanthomas or corneal arcus, which may be present; therefore, in the absence of testing, points will be lost. Finally, even a single point lost due to ignorance can result in a lower DLCN score and, occasionally, in a lower probability category, causing impaired risk stratification or even refraining from familial cascade screening. On the other hand, molecular diagnosis is made by genetic testing, which is often a less employed diagnostic strategy.

Despite its clear diagnostic methods and relatively high prevalence, FH is vastly underdiagnosed and underestimated [4, 11]. Although interest in FH has recently increased substantially, there is still a widespread lack of awareness about this disease [12, 13]. However, countries that have implemented screening programmes have reported higher numbers of identified patients [14–16]. A few different methods and algorithms could be implemented; however, opinions on which of them would be the most beneficial still differ widely. It is important that national screening programmes be able to detect not only patients who are already displaying symptoms but also those whose disease is still silent since the early start of lipid-lowering therapy, and the modification of parallel cardiovascular risk factors is key in preventing major cardiovascular events and saving years of life. The lack of screening programmes is particularly evident in Eastern Europe, as no country, to the best of the knowledge, has implemented a national screening programme in this region. In Lithuania, screening for FH is mainly based on an opportunistic testing approach guided by increased LDL-C levels, followed by cascade screening of the first-degree relatives of the detected index cases [7]. The detailed algorithm used for FH detection and screening in the Lithuanian population is described in this paper, together with very first results on the prevalence of CAD among the FH population and subgroups. This is the first algorithm implemented for FH detection in Lithuania, and it is an important step toward better care of FH patients in the country. This algorithm is believed to result in a significantly increased number of screened patients, particularly at a younger age, with a greater probability of receiving timely treatment and better disease-related management. Additionally, the FH screening programme provides invaluable data on the general understanding of FH, allows the identification of gaps in knowledge, and helps to increase awareness among the medical community, which will inevitably improve the care of FH patients.

## Materials and methods

The Lithuanian national FH screening programme was implemented in 2016 and was created on the basis of the Lithuanian High Cardiovascular Risk (LitHir) primary prevention programme [7, 17]. Since 2018, selected patients who signed informed consent forms have been included in the Lithuanian long-term FH observation programme, as well as in the European Atherosclerosis Society Familial Hypercholesterolemia Studies Collaboration (EAS FHSC) global registry [18].

### Selection of patients

A detailed description of FH detection strategies in Lithuania has been published previously [7]. Ultimately, patients with an FH-like phenotype are referred to a specialized lipidology unit by several further described approaches. First, all patients with severe dyslipidemia, defined as LDL-C > = 5 mmol/L, were referred to lipidology centers by any specialist in any link of the health care system. Although general practitioners (GPs) play the most important role in presenting patients, many patients with suspected FH are referred by other specialists, such as cardiologists and intensive care unit specialists when premature CVD is detected, dermatologists if xanthelasmas are suspected, orthopedic surgeons if tendon xanthomas are detected, or even ophthalmologists if premature lipoid arcus is identified. Moreover, patients may be referred by other specialists who suspect or diagnose premature atherosclerosis in locations other than the coronary arteries, for example, vascular surgeons when peripheral artery disease is diagnosed. Furthermore, if the patient is a participant in the LitHir programme and whose lipid profile reveals dyslipidemia with LDL-C levels above 4,9 mmol or if the patient is at high cardiovascular risk (according to European Society of Cardiology guidelines), they are referred (mostly by primary care physicians (GPs)) for a lipidologist consultation. Participation in the LitHir programme is available for all Lithuanian citizens who have reached predetermined ages—40–55 years for males and 50–65 years for females—since the end of 2023—for all Lithuanian citizens aged between 40 and 60 years. Finally, FH in children and adolescents is detected mostly by cascade screening, as well as if they are diagnosed with premature and/or severe forms of dyslipidemia in pediatric centres. While DLCN criteria for FH diagnosis are not suitable for children, the diagnosis of FH in the pediatric population is based on elevated LDL-C (> 3.9 mmol/l) and a first-degree relative’s history of FH or premature CAD. These patients are consulted and treated by pediatricians or pediatric cardiologists working at the Coordinating Centre of Rare Diseases in Vilnius University Hospital Santaros Klinikos.

### Examination of the potential FH patient

Patients with an FH-like phenotype are being consulted in a specialized lipidology unit. The visit comprises medical data collection, physical examination, laboratory testing, imaging, and instrumental tests. Above all, a very detailed medical history was collected, including personal and familial anamnesis of known FH cases, history of dyslipidemia, cardiovascular diseases and events, and other medical and social anamnesis. Physical examination is directed toward detecting xanthelasmas and measuring and evaluating blood pressure, heart rate, body weight, waist circumference and the general condition of the patient. A search for pathognomonic signs of the FH is also conducted. Patients are referred to an ophthalmologist in search of a lipoid corneal arcus. However, it is worth noting that patients are offered consultation if at their baseline visit, they are younger than 45 years. Finally, patients are referred to a radiologist for ultrasonographic evaluation of the Achilles tendon to detect tendon xanthomas.

The complete lipid profile that is being evaluated includes total cholesterol (TC), low-density lipoprotein cholesterol (LDL-C), high-density lipoprotein cholesterol (HDL-C) and triglyceride levels; apolipoprotein A1; apolipoprotein B (the apoB/ApoA1 ratio); apolipoprotein E; lipoprotein A (Lp(a)); and lipoprotein electrophoresis. Other laboratory tests were performed to rule out secondary causes of dyslipidemia. First, renal function tests (creatinine and estimated glomerular filtration rate) are being performed to rule out dyslipidemia caused by nephrotic syndrome. Additionally, urinalysis is being performed in search of microalbuminuria. Hepatic function tests (aspartate aminotransferase (AST), alanine transferase (ALT), alkaline phosphatase (ALP), gamma glutamyl transferase (GGT)) are being performed to exclude hepatic function impairment and to assess the condition of the liver. Thyroid function is evaluated by thyrotropin (TTH) levels to exclude hypothyroidism, which is one of the most common causes of secondary hypercholesterolemia. Furthermore, plasma glucose and glycosylated hemoglobin levels are measured to exclude diabetes mellitus (DM) (or glucose tolerance impairment), detect metabolic syndrome, and evaluate the control of blood glucose levels in patients with previously diagnosed DM, as poor control of diabetes results in increased LDL-C levels. Finally, high-sensitivity C-reactive protein (hsCRP) levels are measured to aid in cardiovascular risk stratification.

Instrumental testing plays a particularly important role in the examination of FH patients. First, a 12-lead electrocardiogram (ECG) and echocardiography are being performed for general cardiac evaluation, as well as for assessing possible heart rhythm and conduction disorders and functional or structural damage of the heart, with particular attention given to the aortic valve. Patients are also referred for multiple tests that evaluate vascular markers of early atherosclerosis, including measurement of intima-media thickness with detection of atherosclerotic plaques in the common carotid artery, carotid-femoral pulse-wave velocity determination, evaluation of endothelial function using flow-mediated dilatation assessment, and measurement of the cardio-ankle vascular index and ankle-brachial index. These tests are being performed to facilitate cardiovascular risk stratification.

### Diagnosis of FH and initiation of cascade screening

Subsequently, clinical diagnosis is made according to the DLCN criteria, and each patient is categorized as unlikely, possible, probable, or definite FH. If the patient matches the criteria for probable or definite FH and/or is noted to have LDL-C equal to or greater than 6.5 mmol/l, genetic testing and cascade screening of first-degree relatives are initiated. Genetic testing is performed from a dried blood spot. Genomic deoxyribonucleic acid (DNA) is enzymatically fragmented, and regions of interest are enriched using DNA capture probes. The final indexed libraries were sequenced on an Illumina platform (next-generation sequencing). Several selected patients were invited to participate in further monitoring and follow-up. Those who signed an informed consent form were enrolled in the Lithuanian long-term FH observation programme, and their medical data (relevant to FH) were entered into the EAS FHSC international registry.

### Monitoring and follow-up of FH patients

All patients included in the long-term FH observation programme were invited for follow-up visits according to the protocol. The first follow-up visit was 4–6 weeks after the baseline visit, the second 3 months after the baseline visit, and the third 6 months after the baseline visit. Other visits are yearly. The purpose of the follow-up is to monitor the treatment (its effectiveness and possible side effects) and condition of the patient (cardiovascular risk, coronary events, and general condition). Therefore, a full lipid profile was obtained at every visit, along with liver enzyme test and fasting blood glucose data. If the patient had complaints related to the muscular system, the creatine kinase (CK) test was added to the standard tests.

### Statistical analysis

All the statistical analyses were performed using the R (v. 4.0.4) program package. To test hypotheses for between-two-group comparisons of the quantitative variables, Student’s t test or the nonparametric Mann‒Whitney U test was used as appropriate. To test hypotheses for comparisons of quantitative variables between more than two groups, one-way analysis of variance (ANOVA) or the nonparametric Kruskal‒Wallis test was used as appropriate. Normality was tested using the Shapiro‒Wilk test. To test hypotheses for between-group comparisons of categorical variables, Pearson’s chi-square test or Fisher’s exact test was used as appropriate. A *P* value less than 0.05 indicated statistical significance.

## Results

**Fig. 1:**
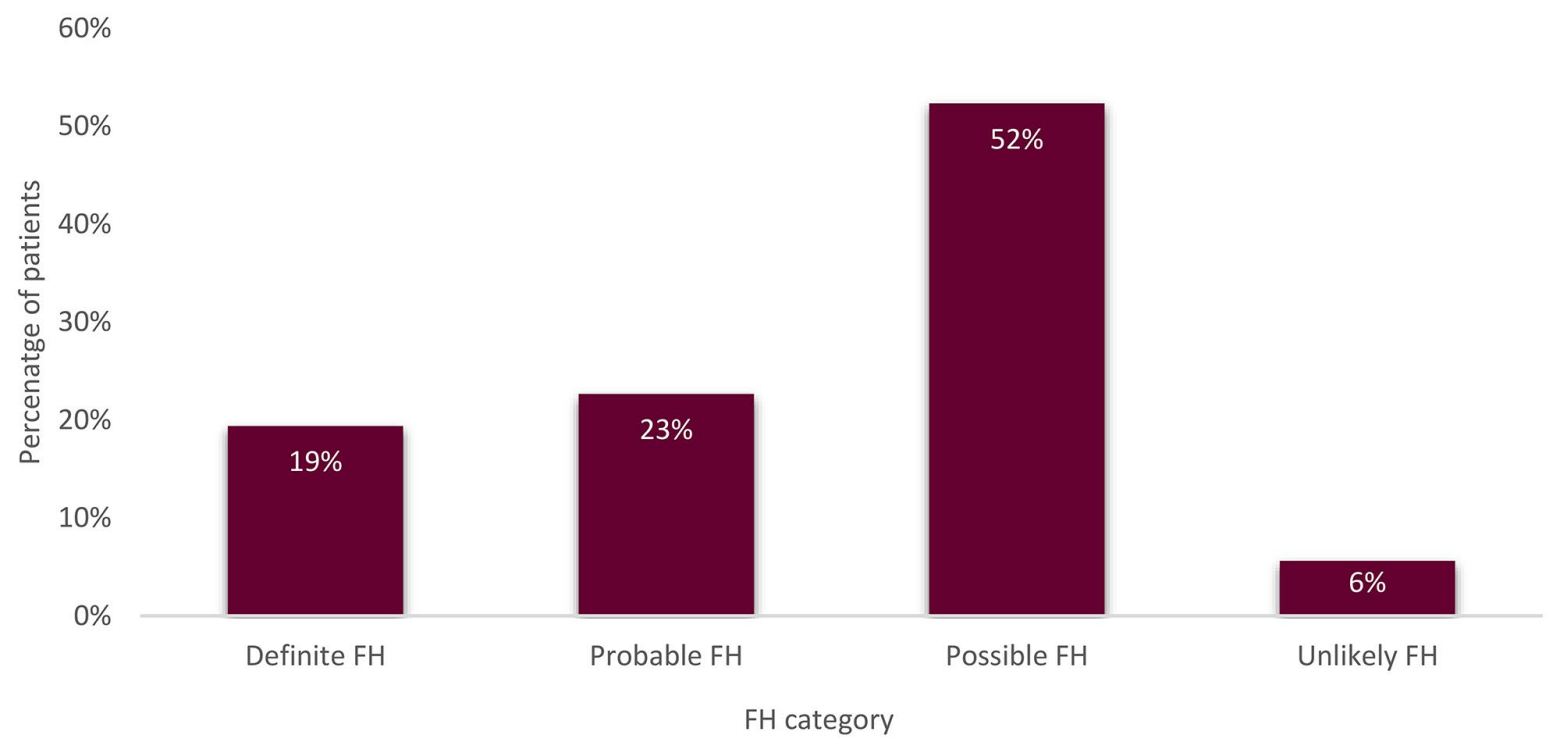
Distribution of DLCN categories among all patients. FH – familial hypercholesterolemia; DLCN – Dutch Lipid Clinic Network criteria

A total of 428 patients were included in the Lithuanian long-term FH observation programme and the EAS FHSC registry. The sample consisted of 228 females (53%) and 200 males (47%). The overall median age at FH diagnosis was 47 (± 11,8) years: 43 (± 10,4) years for males and 53 (± 12) years for females (*P* < 0.005). Based on the DLCN criteria, 224 (52%) patients were classified as having a possible FH diagnosis, 97 (23%) had probable FH, 83 (19%) had definite FH, and 24 (6%) patients were included in the unlikely FH DLCN category (Fig. 1). There were no statistically significant differences in DLCN distribution between the sexes (*P* > 0.5).

The median LDL-C level within this study sample was 6.37 mmol/l (± 1.63). The highest recorded LDL-C in this study was 20.52 mmol/l, whereas the lowest was 2.51 mmol/l. A total of 127 (30%) patients were genetically tested. FH-related mutations were found in 38.6% (*n* = 49) of the patients, while no mutations were detected in 61.4% (*n* = 78) of the genetically tested subjects. Among the patients in whom the genetic mutation was identified (*n* = 49), 27 (55.1%) had a mutation in the LDLR gene, 15 (30.6%) had a mutation in the APOB gene, and one had a mutation in both the LDLR and APOB genes. One patient was found to be homo(hemi-)zygous for the low-density lipoprotein receptor adaptor protein 1 (LDLRAP1) gene variant, causing extremely rare autosomal recessive hypercholesterolemia (ARH). This case was described in a previous publication [19]. For the remaining six patients, data on which gene the mutation was located were not available. All patients in this study, except the one with ARH, presented with the heterozygous FH type.

Coronary artery disease (CAD) was diagnosed in 13% (*n* = 57) of the included patients, whereas premature CAD was found in 47 (11%) patients. Of these 47 patients, 10 (21%) were in the possible FH DLCN category, 15 (32%) had definite FH, and 22 (47%) were in the probable FH DLCN category.

**Fig. 2:**
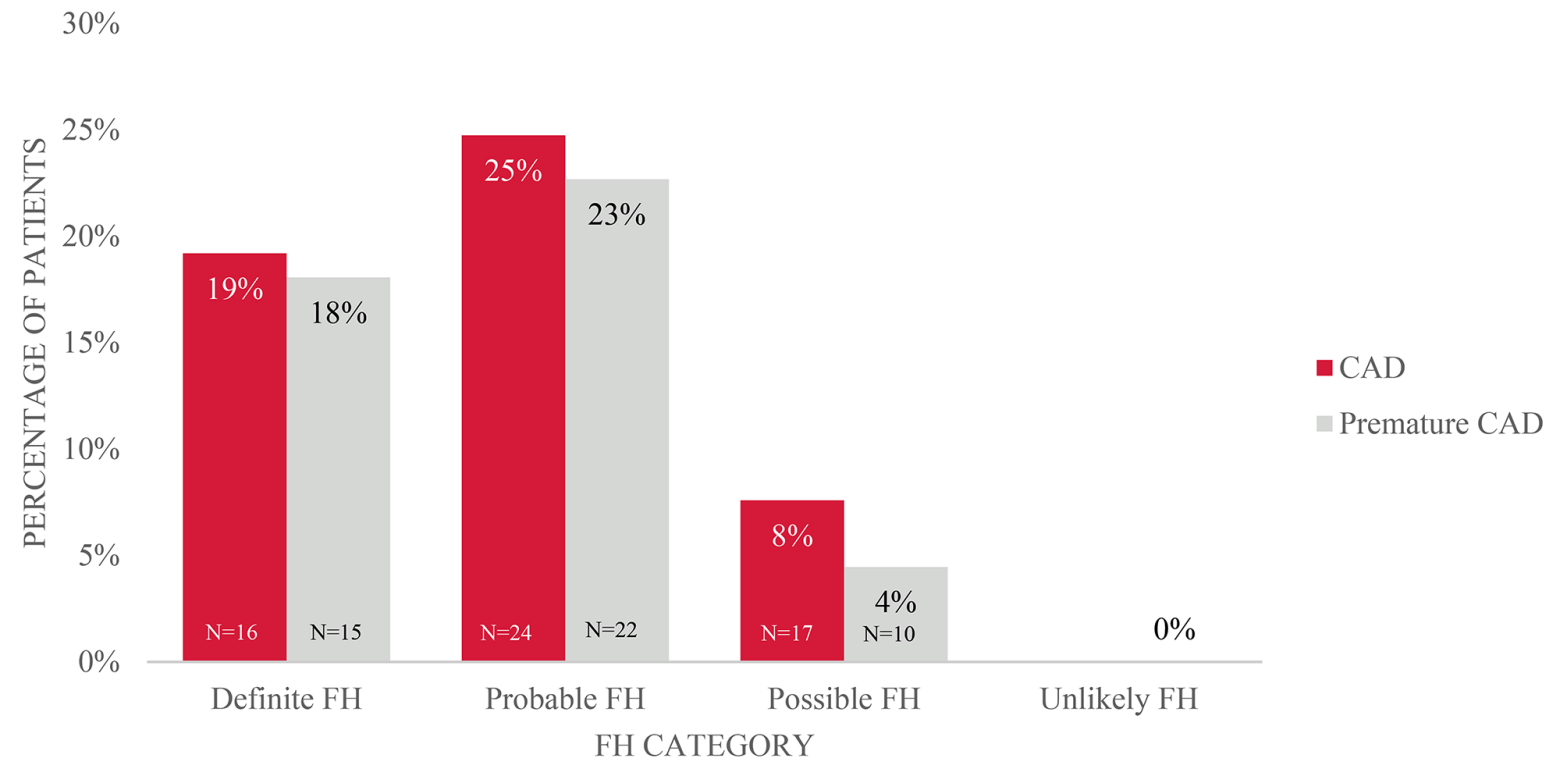
Occurrence of CAD and premature CAD among patient groups according to DLCN category. FH ? familial hypercholesterolemia; DLCN ? Dutch Lipid Clinic Network criteria; CAD ? coronary artery disease

The distributions of CAD and premature CAD according to DLCN category are presented in Fig. 2. In the possible FH diagnosis group, 17 patients (8%) had coronary artery disease (CAD), and for 10 (4.46%) of these patients, the disease was considered premature. In the definite FH group, 16 (19.2%) patients had CAD, 15 (18.07%) of whom had premature CAD. In the probable FH group, 24 (25%) patients had CAD, 22 (22.68%) of whom had premature CAD. None of the patients in the unlikely FH group had CAD at baseline. All the groups differed significantly from each other (*P* < 0.05).

**Fig. 3:**
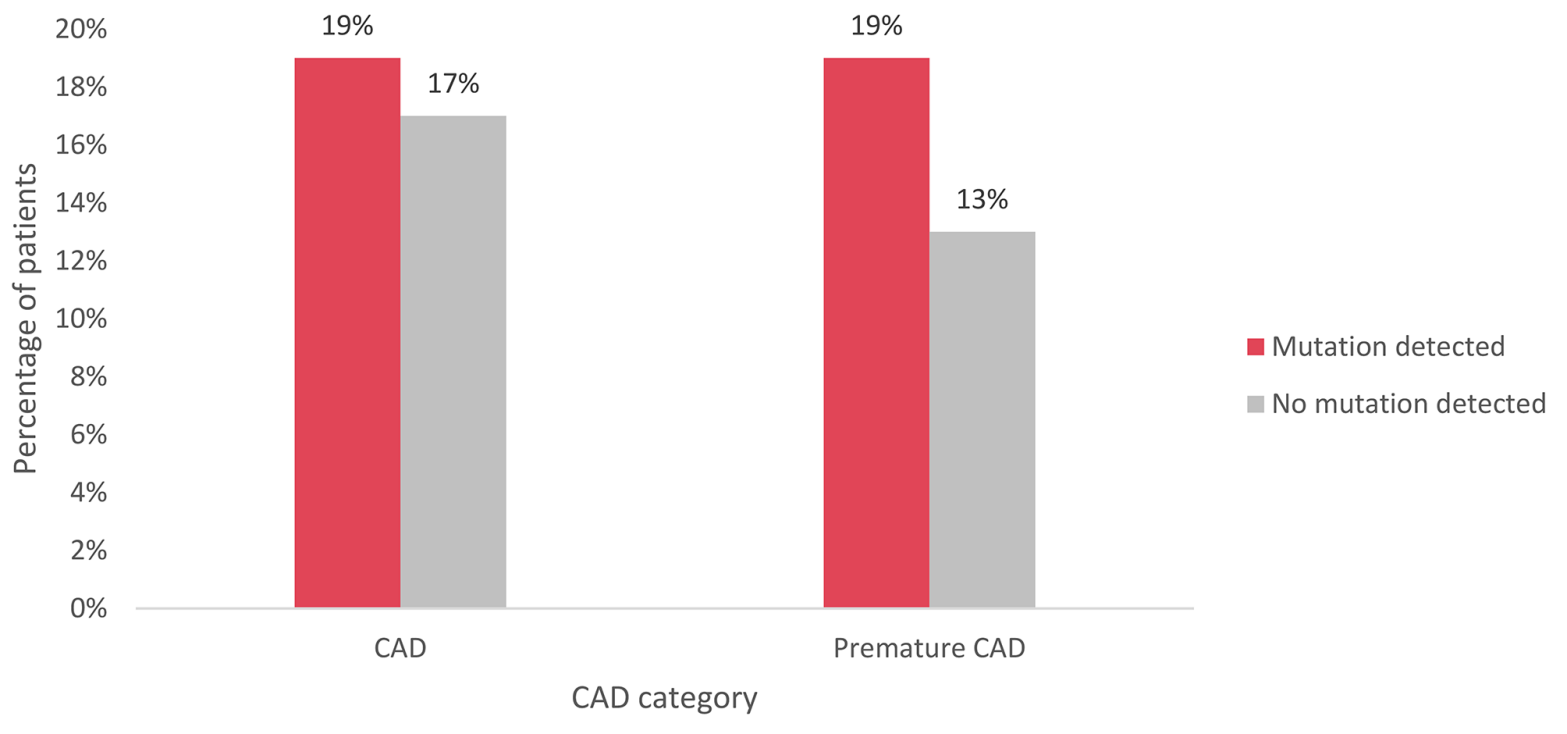
Occurrence of CAD and premature CAD among patient groups according to FH-causing mutation status. FH – familial hypercholesterolemia; CAD – coronary artery disease

**Fig. 4:**
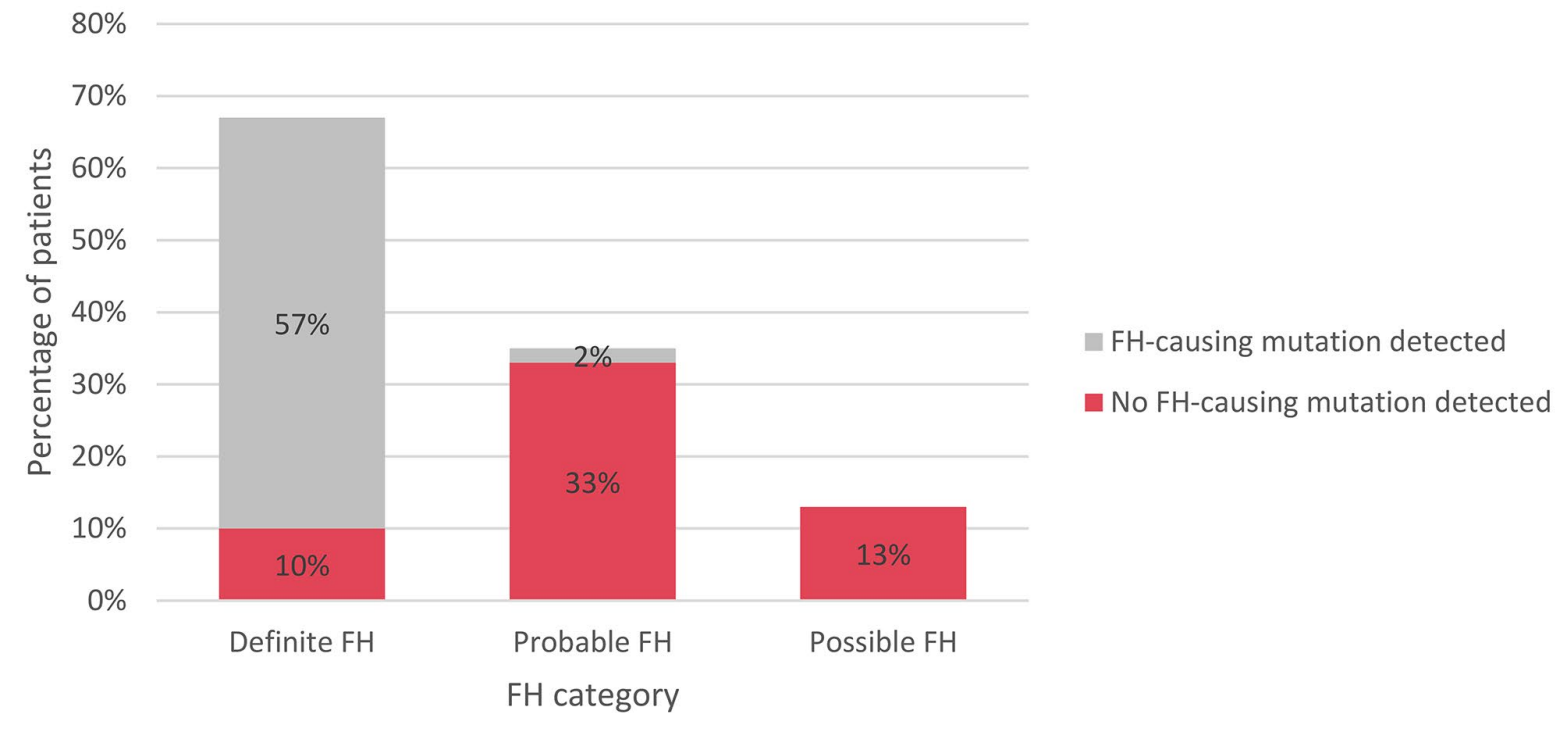
Distribution of mutation status among different FH categories according to DLCN. FH–﻿ familial hypercholesterolemia; DLCN–﻿ Dutch Lipid Clinic Network criteria;

The occurrence of CAD and premature CAD was analyzed among patients (*n* = 125) with FH-related mutations (*n* = 48) and those without mutations (*n* = 77) (Fig. 3 and Fig. 4). CAD was diagnosed in 19% (*n* = 9) of patients with FH-related mutations; of note, it was premature for all these patients. CAD was diagnosed in 17% (*n* = 13) of patients without a mutation, and in 13% (*n* = 10) of these patients, it was determined to be premature CAD. The difference in premature CAD occurrence between the two groups (with and without an FH-causing mutation) was not statistically significant (*P* = 0,383).

**Fig. 5:**
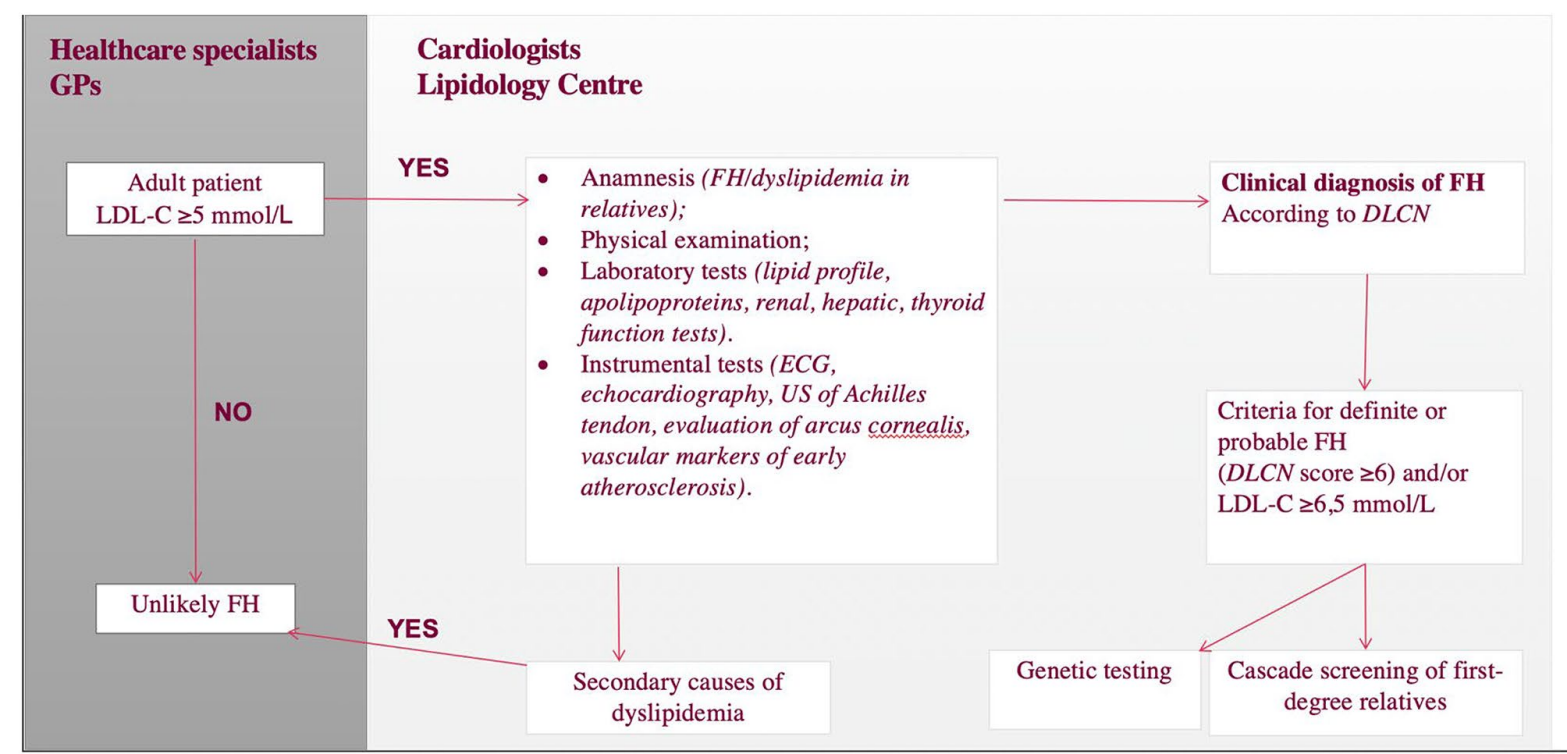
A schematic representation of the FH detection algorithm presented in this study. FH – familial hypercholesterolemia; DLCN – Dutch Lipid Clinic Network criteria; GPs – general practitioners, US – ultrasound, ECG – electrocardiogram

## Discussion

This paper presents the algorithm used in Lithuania to detect FH patients and initiate further screening. FH is now widely recognized as a public health care issue. Early detection and aggressive timely treatment of FH are highly important for preventing ASCVD caused by permanent exposure to increased LDL-C blood levels [20]. The currently used screening strategy in Lithuania is mainly based on opportunistic testing guided by increased LDL-C levels, followed by cascade screening of first-degree relatives when an index case of FH is detected. Cascade screening is the most commonly used FH screening model worldwide [21, 22]. The Lithuanian High Cardiovascular Risk (LitHir) primary prevention programme enables us to opportunistically access approximately 46% of Lithuanian middle-aged citizens every year and evaluate their cardiovascular risk. Therefore, LitHir provides a noteworthy possibility to detect a high percentage of patients with an FH-like phenotype who would otherwise most likely stay asymptomatic until a manifestation of a cardiovascular event. Studies have shown that FH causes atherosclerotic changes in the cardiovascular system as early as childhood, which further highlights the importance of early detection of FH [23]. In this study, the median age at FH diagnosis was 47 years, and 13% of the included patients were diagnosed with coronary artery disease, which is nearly twice the prevalence of CAD in the general Lithuanian population, which was 6.97% in 2022, as estimated based on data available from the Lithuanian Health Education and Disease Prevention Centre [24]. In contrast, in the The Copenhagen General Population Study, among the patients with FH, prevalence of CAD was found to be as high as 33% [25]. Concerning the high median age of FH diagnosis, findings from this study are compatible with those from other studies, especially as *deGoma et al.* in their study on CASCADE-FH Registry also found the median age of FH diagnosis to be 47 years [26]. At that point, at least some of the atherosclerotic damage may be irreversible. However, it has been proven that with adequate treatment beginning at an early age, the cardiovascular risk for FH patients may decrease to a level similar to that of the general population [27]. Furthermore, compared with men, women were significantly older at the time of FH diagnosis, with a median age at diagnosis difference of 10 years. One possible explanation is that the LitHir programme was available for women at a later age (from 50 to 65 years old) than for men (from 40 to 55 years old); in that way, it was biased against women, as it may have caused a delay in adequate treatment of FH. Since the end of 2023, this programme has been available for all Lithuanian citizens aged between 40 and 60 years. In addition, as the goal of FH screening is to prevent health impairment caused by dyslipidemia, an ideal screening program should also be focused on detecting FH patients before constant exposure to increased blood LDL-C levels occurs.

In this regard, universal screening for FH in children combined with cascade screening of first-degree relatives would probably be the most appealing model. However, the cost-effectiveness of universal FH screening is still controversial. Implementing a national universal screening model for FH is complicated, as the exact age at which children should be tested is uncertain—although FH may start affecting the cardiovascular system at an early age, unfortunately, neonatal testing for FH is not possible due to multiple factors affecting neonatal TC and LDL-C blood levels [5]. On the other hand, Slovenia is currently the only country that has implemented universal testing of children—they had success in testing preschool children at the age of 5 [22].

GPs are, in most cases, responsible for the first step of FH detection and should not be overlooked. Since cascade screening relies on index case detection, this algorithm is heavily dependent on the first medical contact (mostly GPs) performing and evaluating patients’ lipid profiles. However, several studies show that GPs across the world lack knowledge about FH and are frequently not aware of current guidelines about dyslipidemia management and cascade screening recommendations [12, 13]. Such gaps in GPs’ knowledge about FH may contribute to both the underdiagnosis and undertreatment of FH. Studies defining the situation in northeastern Europe as well as interventions to raise awareness of FH for not only specialists in lipidology but also GPs are needed.

Since multiple FH search strategies are employed in Lithuania, a great number of patients are screened for FH and consequently referred and consulted at the lipidology center (Fig. 5). As mentioned previously, FH is characterized by accelerated development of atherosclerosis, which eventually leads to (premature) CVD. Therefore, methodical in-depth evaluation of patients with an FH-like phenotype in tertiary lipidology centers is a key part of this screening program. The specialized lipidology unit is advantageous for patients for multiple reasons. First, the centralization of patients provides the opportunity to create a large database that encompasses real-world data about FH, which will undoubtedly improve the understanding of FH. Moreover, all tests and consultations required for both diagnosis and risk stratification can be performed in one location, with experienced specialists interpreting them. Some tests, which are a part of this algorithm, are not available in smaller outpatient settings. For example, echocardiography is of utmost importance in the evaluation of patients with FH since it has been proven that FH is associated with a greater incidence of aortic valve stenosis [28, 29]. Furthermore, vascular markers of early atherosclerosis, which are not routinely detected in most other healthcare institutions, are used for cardiovascular risk stratification, and a detailed analysis of these markers in the FH population in this study has been published previously [30].

Patients with FH are at increased risk of IHD and premature cardiovascular events. In this study, the DLCN category of clinical FH diagnosis was significantly related to CAD and premature CAD. However, recent findings from other studies suggest, that DLCN may not be an optimal predicting tool of CAD in FH [31]. On the other hand, the identification of the FH-related mutation was not significantly associated with a higher incidence of CAD or premature CAD; however, the tendency toward an association between positive mutation and CAD is evident. Several other studies have concluded that a positive mutation is associated with a greater risk of cardiovascular events [32, 33]. Therefore, the lack of statistical significance could be explained by the relatively small sample size.

It should be noted that this screening algorithm relies on current diagnostic methods for FH, the suitability of which has recently started to be questioned, particularly for the DLCN criteria. In this study, more than half of the analyzed patients were included in the possible FH diagnosis group according to the DLCN criteria, although their phenotype raised strong suspicion for clinicians. Furthermore, clinical and molecular diagnoses do not always correlate. For example, some patients with high DLCN scores may not have genetic mutations. It is possible that currently used tests are unable to detect them or that these patients might have a polygenic form of FH; however, this issue remains. On the other hand, patients with a mutation might have a much milder phenotype than those without a mutation, raising the question of whether genetic testing is necessary in patients with FH. Currently, genetic testing for FH is proven to be a cost-effective diagnostic method, despite common misconception that it is expensive and often limited in availability. A positive genetic test may help clinicians interpret the clinical risk of FH and even increase compliance between the patient and clinician [10].

As mentioned before, data collected from patients who signed a written consent form for enrollment in the Lithuanian FH long-term observation programme were also included in the EAS-FHCS international registry. A recent increase in interest in FH has led to several international systematic projects that collect and process data about FH, which is a major step in creating a better care system for patients with FH. These registries not only motivate FH patients to be monitored but also collect crucial, real-world practical data, highlighting gaps in the diagnosis, management, and follow-up of FH patients, which in turn produces information that will help in educating specialists on how to offer better management for affected patients – an important step forward.

Strengths of the study.

The implementation of a previously described FH screening algorithm allows an increase in the number of detected FH cases not only in the middle-aged Lithuanian population (participants in the LitHir programme) but also among younger patients, including children and adolescents.

The study is mainly based on the LitHir programme, which allows for opportunistic testing of suspected FH cases among a significant part of the middle-aged Lithuanian population. This screening strategy, together with cascade first-degree relatives screening, is believed to be the most cost-effective approach. Furthermore, the prevalence of CAD among different FH groups (clinically and/or molecularly diagnosed FH) has never been previously established nor in Lithuania, neither, to the best of knowledge, in other Eastern European countries.

Finally, the FH patient data gathered in this study are included not only in the Lithuanian long-term FH observation programme but also in the global FH registry (EAS FHSC). Notably, this is the only study from Lithuania participating in this project. This registry is crucial for better understanding country-specific FH peculiarities, reducing gaps in knowledge, and ultimately improving the care of FH patients.

### Limitations of the study

There are several limitations to this study. First, there is still an enormous gap in general awareness about FH among the public and medical communities, and this gap is apparently greater in developing regions of the world, including Lithuania. This leads to a particularly small proportion of the FH population being diagnosed and adequately treated. Although the LitHir programme provides the opportunity to screen a large portion of the Lithuanian middle-aged population, it is also notable that such participation is still not active enough, as it still leaves a large part of the middle-aged Lithuanian population, not to mention the youth, unassessed. Additionally, due to attachment to LitHir, in many cases, this screening model relies heavily on patients’ own interest in their health since participation in LitHir is not obligatory. For most patients with FH, dyslipidemia is “silent” and does not cause any symptoms, which may result in some patients being reluctant to adhere to treatment or start treatment altogether. Unfortunately, despite all the efforts, the availability of genetic testing in Lithuania is still limited, as only 30% of patients were able to be tested for FH-causing mutations. Such barriers to screening should not be overlooked and should be addressed in the future.

## Conclusions

In this study, most patients with suspected FH were classified as probable FH or possible FH according to the DLCN criteria, without differences in age or sex. The median age at the time of FH diagnosis was 47 years, and the FH patients were significantly older females than males, which reflects the strong interface of this study with the LitHir programme. CAD and premature CAD were more common among patients with probable and definite FH, as well as more common among those with an FH-causing mutation; however, this difference was not statistically significant.

This study describes the algorithm used in Lithuania for FH detection and cascade screening initiation. Accurate diagnosis of FH, made by integrating various methods of thorough examination of potential FH patients, helps to exclude secondary causes of dyslipidemia and to select patients for cascade screening initiation more precisely. The algorithm is likely to be the most cost-effective approach for FH screening, as it allows the exact extent of examination required for these patients.

The findings of this study will improve the general understanding of the current situation of FH in Lithuania and will be used to further tailor the algorithm for optimal management of FH patients, as well as maximize the proportion of detected FH cases.

## Data Availability

The datasets used and analysed during the current study are available from the corresponding author on reasonable request.

## Abbreviations

ALP: alkaline phosphatase
ALT: alanine transferase
ANOVA: one-way analysis of variance
ApoA1: apolipoprotein A1
APOB: apolipoprotein B
ApoB: apolipoprotein B
ARH: autosomal recessive hypercholesterolemia
ASCVD: atherosclerotic cardiovascular disease
AST: aspartate aminotransferase
CAD: coronary artery disease
CK: creatine kinase
DLCN: Dutch Lipid Clinic Network
DM: diabetes mellitus
DNA: deoxyribonucleic acid
EAS FHSC: European Atherosclerosis Society Familial Hypercholesterolemia Studies Collaboration
ECG: electrocardiogram
FH: Familial hypercholesterolemia
GGT: gamma glutamyl transferase
GP: general practitioner
HDL: C-high-density lipoprotein cholesterol
HeFH: heterozygous familial hypercholesterolemia
HoFH: homozygous familial hypercholesterolemia
hsCRP: high-sensitivity C-reactive protein
IHD: ischemic heart disease
LDL: C-low-density lipoprotein cholesterol
LDLR: low-density lipoprotein receptor
LDLRAP1: low-density lipoprotein receptor adaptor protein 1
LitHir: Lithuanian High Cardiovascular Risk
Lp(a): lipoprotein A
PCSK9: pro-protein convertase subtilisin/kexin 9
TC: total cholesterol
TTH: thyrotropin
